# Sex differences in insular cortex gyri responses to a brief static handgrip challenge

**DOI:** 10.1186/s13293-017-0135-9

**Published:** 2017-04-20

**Authors:** Paul M. Macey, Nicholas S. Rieken, Jennifer A. Ogren, Katherine E. Macey, Rajesh Kumar, Ronald M. Harper

**Affiliations:** 1 UCLA School of Nursing, University of California at Los Angeles, 700 Tiverton Avenue, Los Angeles, CA 90095-1702 USA; 2 Brain Research Institute, David Geffen School of Medicine at UCLA, University of California at Los Angeles, Los Angeles, CA 90095 USA; 3 Department of Neurobiology, David Geffen School of Medicine at UCLA, University of California at Los Angeles, Los Angeles, CA 90095 USA; 4 Department of Anesthesiology, David Geffen School of Medicine at UCLA, University of California at Los Angeles, Los Angeles, CA 90095 USA; 5 Department of Radiological Sciences, David Geffen School of Medicine at UCLA, University of California at Los Angeles, Los Angeles, CA 90095 USA

**Keywords:** Parasympathetic, fMRI, Cardiovascular, Functional neuroanatomy, Limbic

## Abstract

**Background:**

Cardiovascular disease varies between sexes, suggesting male-female autonomic control differences. Insular gyri help coordinate autonomic regulation and show a sex-dependent response to a sympathetic challenge.

**Methods:**

We examined sex-related insular gyral responses to a short static handgrip exercise challenge eliciting parasympathetic withdrawal with functional magnetic resonance imaging (fMRI) during four 16-s challenges (80% maximum strength) in 23 healthy females (age; mean ± std 50 ± 8 years) and 40 males (46 ± 9 years). Heart rate (HR) and fMRI signals were compared with repeated measures ANOVA (*P* < 0.05). Additional analyses were performed with age and age interactions, as well as right-handed only subjects.

**Results:**

Females showed higher resting HR than males, but smaller percent HR change increases to the challenges. All gyri showed fMRI patterns concurrent with an HR peak and decline to baseline. fMRI signals followed an anterior-posterior organization in both sexes, but lateralization varied by gyri and sex. All subjects showed greater signals in the anterior vs. posterior gyri (females 0.3%, males 0.15%). The middle gyri showed no lateralization in females but left-sided dominance in males (0.1%). The posterior gyri showed greater left than right activation in both sexes. The anterior-most gyri exhibited a prominent sex difference, with females showing a greater right-sided activation (0.2%) vs. males displaying a greater left-sided activation (0.15%). Age and handedness affected a minority of findings but did not alter the overall pattern of results.

**Conclusions:**

The anterior insula plays a greater role in cardiovascular regulation than posterior areas during a predominantly parasympathetic withdrawal challenge, with opposite lateralization between sexes. In females, the left anterior-most gyrus responded distinctly from other regions than males. Those sex-specific structural and functional brain patterns may contribute over time to variations in cardiovascular disease between the sexes.

**Electronic supplementary material:**

The online version of this article (doi:10.1186/s13293-017-0135-9) contains supplementary material, which is available to authorized users.

## Background

Females and males differ in autonomic characteristics ([1–8], for review, see [9]). The processes underlying such differences include physical characteristics, hormone levels, and variations in organization of central regulation of autonomic outflow. One region that is both key to autonomic regulation and shows sex differences in anatomic structure and functional responses to autonomic challenges is the insular cortex. The insular cortex processes autonomic stimuli and regulates autonomic outflow via projections to the hypothalamus and brainstem sites [10–17]. In animals, insular regulation of autonomic functions is region specific, and this regulation appears dynamic but not necessarily tonic [18]. The human insula also activates in response to blood pressure challenges [19–21] in a region-specific manner [22].

The insular cortex usually comprises five main gyri [12], with the anterior-most gyrus, the anterior short gyrus (ASG), most active in response to a sympathetic challenge. Several autonomic challenges elicit this anterior insula-dominant activation, including the static handgrip exercise, cold pressor, and Valsalva maneuver [22]. We previously found sex differences specifically in the right ASG responses to the Valsalva maneuver: in females, this area responded less to a Valsalva than the other anterior short gyri [23]. Furthermore, in contrast with other insular regions in females and males, the right ASG showed a lower response on the right over the left side. This sex-specific altered insular response may be a characteristic of sympathetic activation, as occurs during the Valsalva maneuver, or may be coincident with any heart rate increase. The present study follows from the Valsalva findings to assess responses to a brief static handgrip exercise challenge, which increases blood pressure and heart rate, most likely through vagal withdrawal, without notable increase in sympathetic activity as measured by muscle sympathetic nerve activity (MSNA), at least during brief (<30 s) contractions [21, 24]. Differentiating which arm of the autonomic nervous system, sympathetic or parasympathetic, plays dominant roles in challenges is a significant issue in formulating care for pathologic cardiovascular and other conditions where expression of symptoms may substantially differ between males and females. Brain areas serving those separate components may be subject to injury or otherwise affected between sexes in the pathologic conditions, and interventions must consider those possibilities.

A static handgrip exercise involves gripping forcefully for a period of time and is accompanied by blood pressure and heart rate increases during the first minute [24]. The protocol typically involves maintaining a static grip with a force of 30–100% of maximum grip strength [25]. The static handgrip exercise elicits a cardiovascular pressor response, but in contrast to the Valsalva maneuver, it does not alter thoracic pressure and increase MSNA during the first 30 s [26]. The static handgrip exercise challenge is therefore considered to elicit parasympathetic withdrawal rather than sympathetic activation, at least during contractions less than 30 s, in contrast with the Valsalva maneuver. Hypertensive subjects do show MSNA increases 10 s into a static handgrip exercise task [27], so lack of sympathetic activation can be assumed in healthy people but not necessarily in people in disease states. The static handgrip exercise cardiovascular responses arise in response to muscle activity as opposed to only central command, as neuromuscular blockade greatly attenuates the heart rate and blood pressure increases [28]. Animal experiments show that muscle activity triggered by stimulation, that is without central command, leads to blood pressure and heart rate increases [29, 30]. However, Boulton et al. [31] showed that electrically evoked contractions did not evoke an increase in MSNA to the contracting muscle, thus supporting a dominant role for central command in the increase in sympathetic outlaw to the muscle vascular bed. Furthermore, central command does elicit some cardiovascular responses with no change in muscle activity [32, 33]. Over a period of a minute and longer, muscle fatigue emerges and is accompanied by increased sympathetic outflow, as reflected in increased MSNA [34]. Since short-term heart rate increases to the static handgrip exercise appear to be elicited by different mechanisms than sympathetic challenges such as the Valsalva maneuver, the question is whether the sex differences in autonomic neural regulation are also present with a parasympathetic withdrawal response.

Functional magnetic resonance imaging (fMRI) during a static handgrip exercise provides a measure of insular cortex neural responses during the challenge and recovery period, relative to baseline. Protocols can be short (≤1 min), eliciting vasoconstrictive action and increasing blood pressure and heart rate, with MSNA increases appearing after 15 s, or longer, which will elicit metabolic influences after 1 min [24, 26]. Previous studies report sex differences to exercise, including stronger insular cortex activation in males than females during a 30-s static handgrip exercise, more so on the mid-to-anterior right side [35].

The objective was to assess insular organization across gyri, of fMRI responses to a static handgrip exercise challenge, and compare female and male responses. Based on the Valsalva findings, we hypothesized that the right anterior insular gyrus would show altered organization between the sexes and that the females should show a different pattern in this region, compared with other, more posterior regions.

## Methods

### Subjects

We studied 63 healthy adults (age ± std 47.0 ± 9.1 years, range 31–66 years; 40 males, 23 females). All subjects had no history of cerebrovascular disease, myocardial infarction, heart failure, neurological disorders, or mental illness and were not taking cardiovascular or psychotropic medications. Subjects were recruited from the Los Angeles area and did not weigh more than 125 kg or have any metallic or electronic implants; the latter two issues are MRI scanner contraindications. All subjects provided written informed consent, and the research protocol was approved by the Institutional Review Board of UCLA. No subjects were taking exogenous sex hormones (for example, oral contraceptive pills, hormone replacement therapy, or testosterone therapy).

### Static handgrip exercise protocol

The static handgrip exercise protocol consisted of gripping at 80% subjective maximum grip strength for a sequence of four 16-s periods [25]. Such a protocol allows for identification of brain regions initially recruited to respond to the pressor challenge and for repeated tasks to be performed within a single fMRI scanning session. While regulation over a longer period of time (e.g., 2 min at 30% grip strength) would also be of interest, the initial response is critical to maintain adequate perfusion and hence was chosen as the focus of this investigation. We selected an 80% subjective maximum as opposed to 100% as participants were unable to maintain a consistent grip strength at 100% for 16 s. An air-filled plastic bag, connected to a pressure transducer, was placed in the subjects’ right hand. During the practice period, subjects briefly squeezed at 100% strength at least two times and then at 80% for a sustained period of time. A light signal was used to indicate the onset of each grip period. Subjects were instructed to squeeze to maintain the 80% pressure upon seeing the light signal. Subjects practiced the static handgrip exercise maneuver prior to scanning, both outside and supine inside the MRI scanner. At least 30 min of rest (structural scanning) separated the practice from the trial periods. A pressure signal was monitored to verify that all subjects performed the four static handgrip exercise tasks at the correct time.

### Physiologic signals

Cardiac, load pressure, and indicator signals (e.g., light on/off) were recorded with an analog-to-digital acquisition system (instruNet INET-100B, GWI Instruments, Inc., Somerville, MA). Heart rate was assessed using an MRI-compatible pulse oximeter (Nonin Medical Inc., Plymouth, MN). The sensor was placed on the left index finger throughout the scan, and heart rate was calculated from the raw oximetry signal acquired at 1 kHz using custom peak-detection software followed by expert review. Patient cue signals were simultaneously recorded, and all signals were synchronized to the MRI scans, and data corresponding to the fMRI recording period extracted.

### MRI scanning

Functional MRI scans were acquired using a 3.0-T scanner (Siemens Magneton Tim-Trio, Erlangen, Germany), while subjects lay supine. A foam pad was placed on either side of the head to minimize movement. We collected whole-brain images with the blood-oxygen level dependent (BOLD) contrast (repetition time (TR) = 2000 ms; echo time (TE) = 30 ms; flip angle = 90°; matrix size = 64 × 64; field-of-view = 230 × 230 mm; slice thickness = 4.5 mm). The spatial resolution was based on achieving whole-brain coverage, with the fastest possible acquisition time. Two high-resolution T1-weighted anatomical images were also acquired with a magnetization prepared rapid acquisition gradient echo sequence (TR = 2200 ms; TE = 2.2 ms; inversion time = 900 ms; flip angle = 9°; matrix size = 256 × 256; field-of-view = 230 × 230 mm; slice thickness = 1.0 mm). Field map data consisting of phase and magnitude images were collected to allow for correction of distortions due to field inhomogeneities.

### MRI data preprocessing

All anatomical scans were inspected to ensure the absence of visible pathology. For each fMRI series, the global signal was calculated and the images realigned to account for head motion. Subjects with large changes in global BOLD signal or who moved more than 2° or 4 mm in any direction were not included in the study. Each fMRI series was linearly detrended to account for signal drift (but not global effects) [36], corrected for field inhomogeneities, spatially normalized, and smoothed (8-mm Gaussian filter), and mean time trends from each voxel were calculated across all subjects, as well as the challenge means across each of the four static handgrip exercise periods. A mean image of all subjects’ spatially normalized, anatomic scans was created. Software used included the statistical parametric mapping package, SPM12 (Wellcome Department of Cognitive Neurology, UK; www.fil.ion.ucl.ac.uk/spm), MRIcroN [37], and MATLAB-based custom software.

### Region-of-interest tracing

**Fig. 1:**
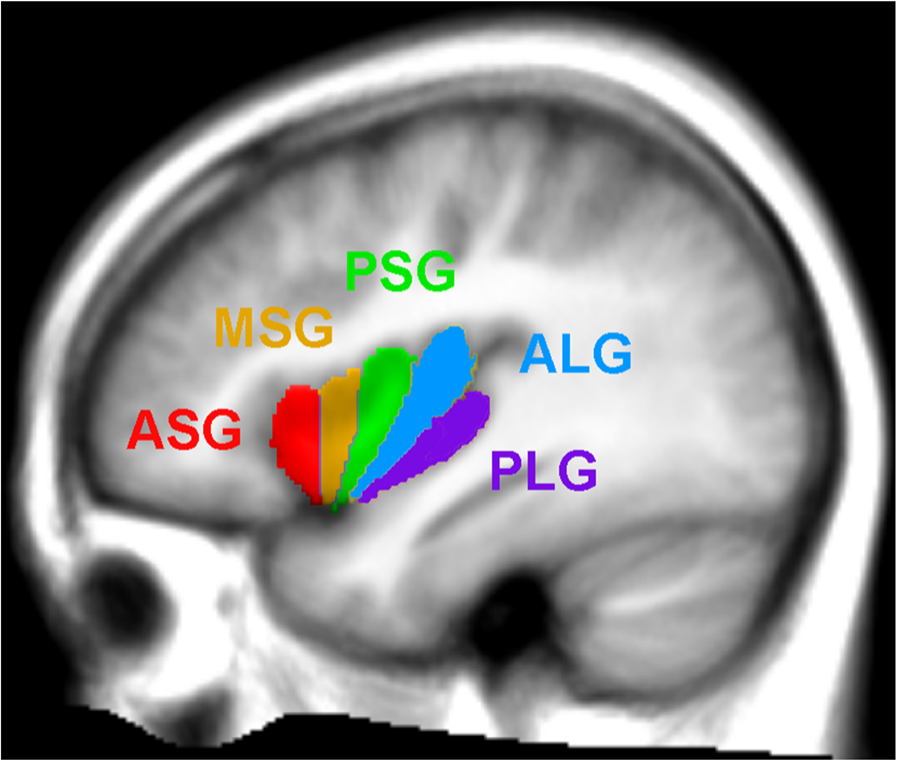
Insular gyri color-coded and overlaid on an average anatomical scan. The anterior region of the insula is comprised of the short gyri, including the anterior short gyrus (*ASG*), mid short gyrus (*MSG*), and posterior short gyrus (*PSG*). The posterior region of the insula is comprised of the long gyri, including the anterior long gyrus (*ALG*) and posterior long gyrus (*PLG*)

The five major gyral regions in the insular cortex, the anterior short gyrus (ASG), mid short gyrus (MSG), posterior short gyrus (PSG), anterior long gyrus (ALG), and posterior long gyrus (PLG), were outlined on the mean anatomical image with MRIcroN software [37], using previously published anatomical descriptions [12, 38]. Figure 1 illustrates the gyri on an average anatomical scan in a sagittal view. While individual tracing would be more accurate for identifying gyral differentiation on anatomical scans, the fMRI data are at a much lower spatial resolution (voxel volume of 53 vs. 0.8 mm^3^ for the anatomical scans), and the BOLD effect itself, which is the basis for assessing neuronal responses, is diffuse, so the advantage of individual tracing would be minimal. Since gyral folding in the insula has individual variation [39], the present approach distinguishes gyral regions rather than gyri per se. The three main gyri of the anterior insula, the ASG, MSG, and PSG, make up the convex surface of the structure and are visible on the sagittal and axial views of the mean anatomical image. The accessory and transverse gyri, two other gyri in the anterior insula, are difficult to visualize [38] and were not visible on the mean anatomical image. Thus, in our tracing of the ASG, we included the entire anterior-most portion of the insula, which included the both accessory and transverse gyri. The posterior gyri (ALG and PLG) were easily visible on sagittal, as well as axial sections of the anatomical volume.

### Statistical analysis

Repeated measures analysis of variance (RMANOVA), implemented with the mixed linear model procedure “proc mixed” in SAS 9.4 software [40], was used to identify periods of significant response relative to baseline, during the static handgrip exercise and subsequent recovery periods [41]. We modeled the fMRI responses as a function of scan time points. Significance was first assessed at the global level; as per the Tukey-Fisher criterion for multiple comparisons, the time points of significant responses were identified only for significant models (*P* < 0.05). Three sets of models were created: (1) within and between group analyses of fMRI signal change relative to baseline; (2) separate female and male between-group signal change relative to PLG, where the categorical variable group consists of five gyri in one hemisphere (with six between-gyri comparisons each); and (3) separate female and male signal change of left relative to right gyri. Statistical assessment of sex differences for model 2 was not performed, as those signals were relative to another gyrus, such that the signal in males was relative to a different reference than females, which would complicate interpretation of RMANOVA-identified female and male differences. To avoid potential confounds due to global vascular effects, we focused on relative changes between gyri. The restriction of only assessing differences rather than absolute responses results from the relative nature of the BOLD-based fMRI technique. To characterize anterior-posterior organization, we assessed responses with respect to the PLG. We choose the PLG as the reference because the posterior insula typically responds less than anterior regions in response to autonomic stimuli [42]. To identify lateral organization, we assessed right-sided relative to left-sided responses for each gyrus [22], that is, right minus left. We did not include a hemisphere factor in any model, since the aims were restricted to identifying gyrus-specific differences.

To summarize, our primary analysis consist of 19 models: 10 for between-sex comparisons in each gyrus, 4 for between-gyri comparisons in each hemisphere and each sex, and 5 for between-sex laterality comparisons in each gyrus.

### Additional analyses: age effects and handedness

Autonomic function changes with age, and our sample was not exactly age matched, so we performed a secondary analysis of the effects of age, including interactions of age with timing of responses and with sex. This step involved creating additional models with age effects, resulting in the following five sets of dependent variables:

1. Original: sex, time, sex × time;
2. Age effect: sex, time, sex × time, age;
3. Age with time interaction: sex, time, sex × time, age, age × time;
4. Age with sex and time interactions: sex, time, sex × time, age, age × sex, age × time;
5. Age with sex interactions: sex, time, sex × time, age, age × sex.

For between-gyri analyses, the categorical factor  “gyrus” replaces “sex” in the above models. The significances of the three effects in the original model were compared with their equivalents in the four age-related models and classified as “same” if the significance did not change from above or below the *P* = 0.05 threshold.

Handedness could influence the grip strength, so right-handed subjects were analyzed separately. For each analysis, the five models were implemented with only right-handed subjects. The significances of the effects in the all-subject models were compared with their equivalents in the right-handed only models and classified as “same” if the significance did not change from above or below the *P* = 0.05 threshold.

## Results and discussion

### Subjects

Of the 63 subjects, the 23 females were, on average, slightly older than males (mean age ± std: female 50.3 ± 7.8 years, male 45.9 ± 9.1 year), although the difference was not significant (*P* > 0.05, independent samples *t* test). Body mass index also did not differ significantly (female 23.9 ± 5.0 kg/m^2^, male 25,2 ± 2.8 kg/m^2^; group difference *P* = 0.2, independent samples *t* test). Seven females reported being left handed, three ambidextrous, and 13 right handed. Five males reported being left handed, two ambidextrous, and 33 right handed. The difference was not significant (*P* = 0.13, chi-square).

### Physiology

**Fig. 2:**
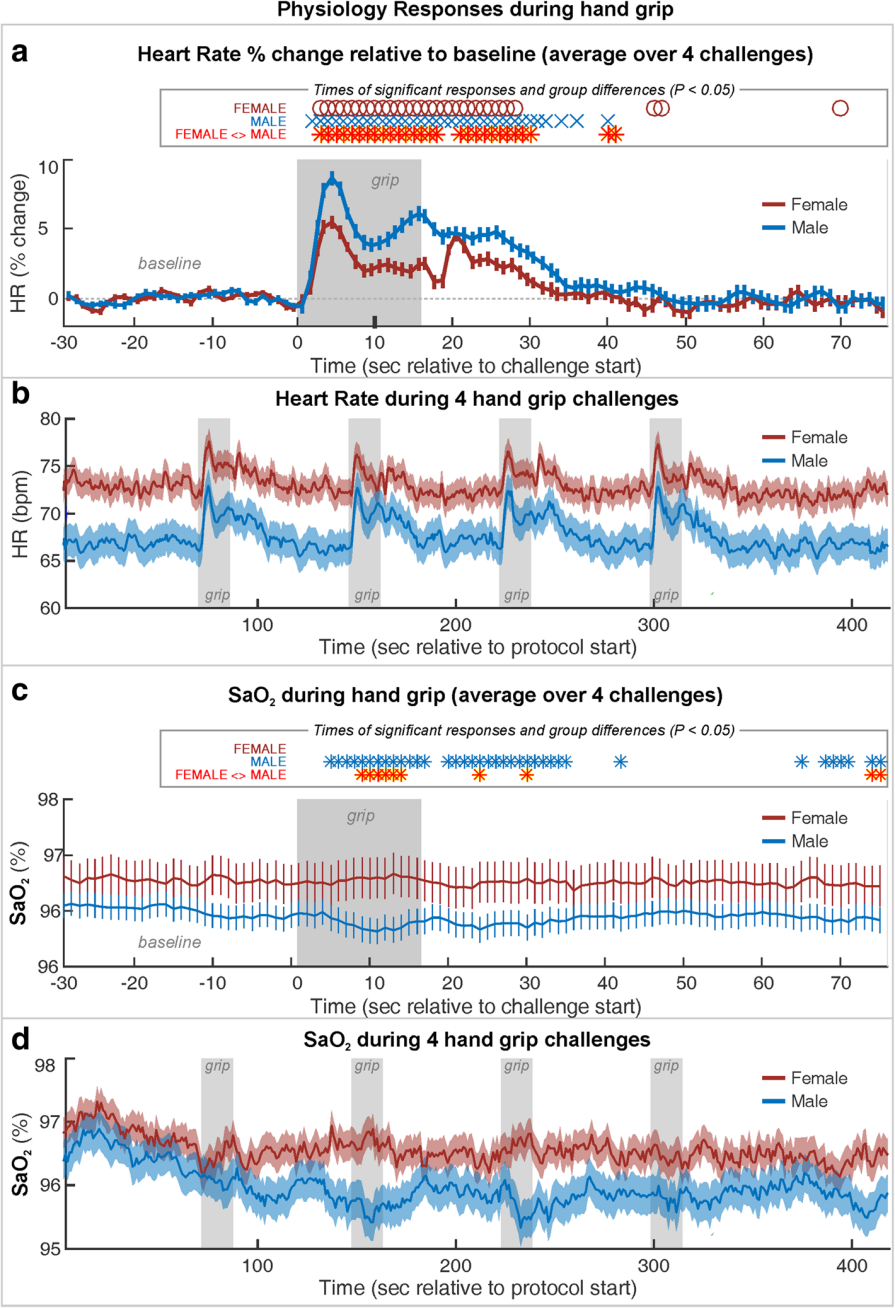
Heart rate (HR) and SaO_2_ changes during a series of four static handgrip exercise challenges, averaged for female and male groups. HR is from all 63 subjects, and SaO_2_ is from 58 subjects with artifact-free mean saturation data. **a** HR % change relative to baseline and **c** SaO_2_, averaged over four challenges (mean ± SE). Time points of significant within-group responses are indicated by *blue xs* (males) and *red circles* (females), and significant between-group differences in *red-yellow asterisks*, based on *P* < 0.05 with repeated measures ANOVA (RMANOVA). **b** Absolute heart rate and **d** SaO_2_ over series of four static handgrip exercise challenges, averaged separately over females and males with SE shaded

Heart rate showed significant sex, time, and sex × time effects (all *P* ≤ 0.001), with relative increases in females and males during the grip period (model statistics: chi-square = 2722, −2 res log-likelihood = 162,589; Fig. 2a). Visual inspection of the trends over the protocol confirms similar responses for each of the four challenges (Fig. 2b). As in other healthy populations, the absolute heart rates were higher in females than males (Fig. 2b). Four females and three males showed artifact in the mean saturation signal at times during the series and were excluded from the SaO_2_ analysis. The remaining 37 males showed a decrease in oxygen saturation (SaO_2_) from 5 s into the challenge, whereas the 19 females showed no significant change in SaO_2_ (Fig. 2c). The response patterns differed between the two groups from 9 to 14 s into the challenge. However, the plot of SaO_2_ over the protocol (Fig. 2d) suggests that the two groups started at a similar level, but the males decreased further during the recovery from the first challenge and then remained at a lower level throughout the remainder of the challenges.

### fMRI responses: sex differences

**Fig. 3:**
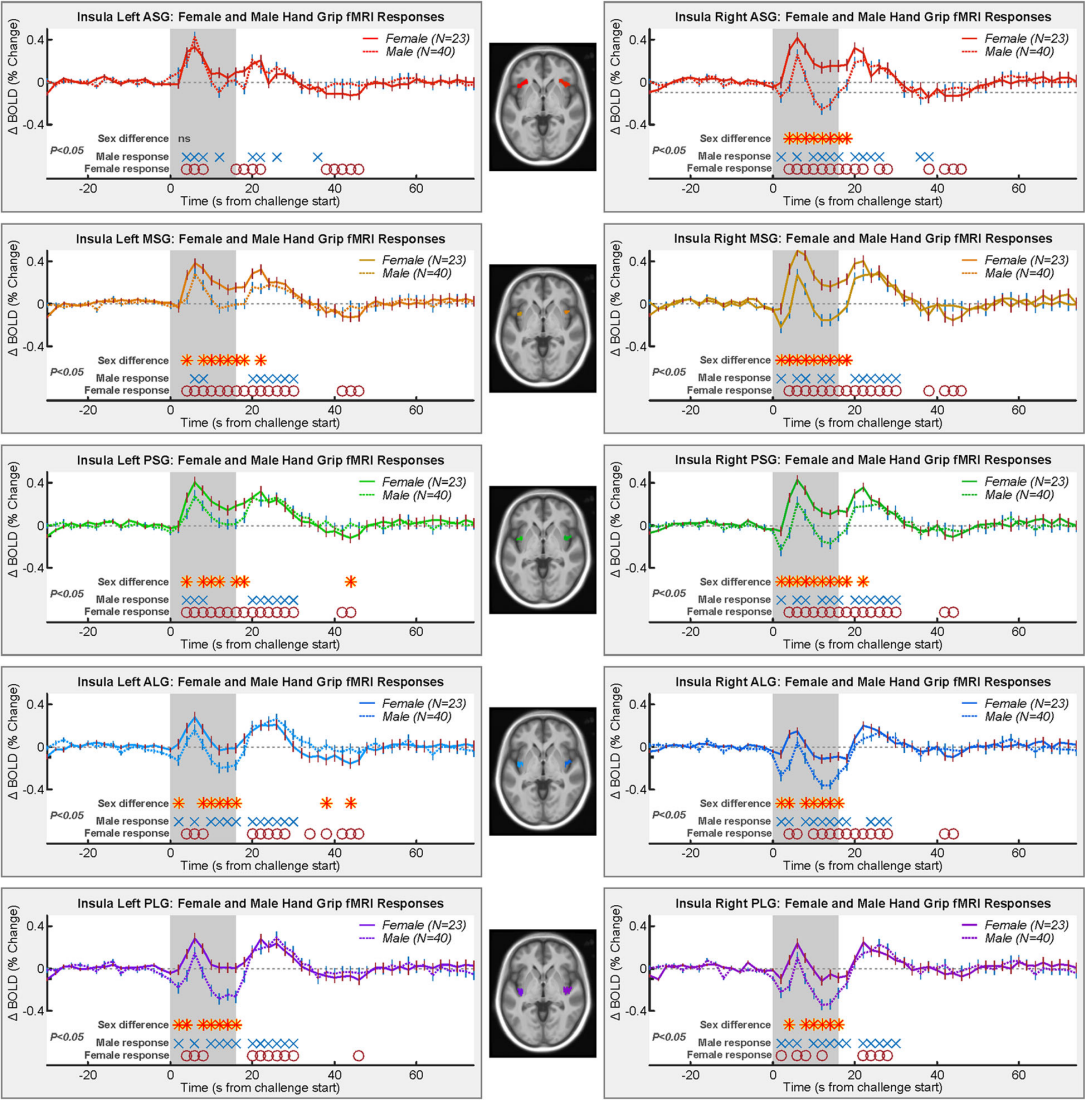
Mean fMRI insula responses over four static handgrip exercise challenges, averaged for female and male groups. All left and right gyri response patterns are shown. Time points of significant within-group responses and between group differences are indicated above the *x*-axis and below the graphs (RMANOVA *P* < 0.05; Table 1)

The static handgrip exercise challenge elicited significant fMRI signal responses that differed from baseline in all insular gyri in both sexes (Fig. 3). For both males and females, neural responses were present during the challenge and recovery periods (blue X’s and red O’s in Fig. 3). Female and male responses differed in all gyri except the left ASG (Table 1; Fig. 3). The responses generally showed an increase, peaking between 5 and 6 s into the challenge, followed by a decrease in the response to a nadir between 12 and 16 s into the challenge and a second peak 4–10 s into the recovery period. While patterns included increases and decreases relative to baseline, the magnitude of female responses was consistently higher than in males during the static handgrip exercise challenge in the gyri where female and male responses differed, as shown by the higher group averages (that is, the solid female lines higher than dashed male lines in Fig. 3). Group differences were present from 4 s into the challenge, shortly after the first static handgrip exercise (*P* < 0.05*,* RMANOVA red-yellow stars in Fig. 3). In the left gyri, the female response initially increased faster than the male response, but the peak response did not differ; however, the male response declined faster and farther than the female response, except in the PSG, where the female trough approached the response from the males and then diverged briefly at the onset of the recovery. In the PSG, the female response briefly dipped lower than the male response 28 s into the recovery period. Females also exhibited lower responses at 38 and 44 s into recovery in the left ALG. In the right gyri, females exhibited a higher response from 2–4 s into the challenge until the end of the challenge, or 2 s into recovery, with the exception of the time of the peak response in the right ALG and PLG where the male response approached the female response. In the right PSG, the secondary peak was higher in females at 6 s into the recovery.

**Table 1:** Female vs. male model fit. Overall model chi-square (ChiSq) was always significant (*P* < 0.0001)

Female vs. male		Sex, time sex × time	Sex, time sex × time
		Left ASG	Right ASG
Model	ChiSq (*P* < 0.0001)	321.1	278.88
	−2 log-likelihood	787.7	1447.1
Effects (*P*)	Sex	0.7762	*0.0091
	Time	*<0.0001	*<0.0001
	Sex × time	0.2109	*<0.0001
		Left MSG	Right MSG
Model	ChiSq (*P* < 0.0001)	362.63	651.88
	−2 log-likelihood	819.7	8158.7
Effects (*P*)	Sex	0.0565	*0.0077
	Time	*<0.0001	*<0.0001
	Sex × time	*0.0011	*<0.0001
		Left PSG	Right PSG
Model	ChiSq (*P* < 0.0001)	497.95	655.47
	−2 log-likelihood	680.7	772.3
Effects (*P*)	Sex	0.2902	*0.0087
	Time	*<0.0001	*<0.0001
	Sex × time	*0.0175	*<0.0001
		Left ALG	Right ALG
Model	ChiSq (*P* < 0.0001)	417.84	590.7
	−2 log-likelihood	895.1	493.8
Effects (*P*)	Sex	0.6822	*0.0194*
	Time	* < 0.0001	*<0.0001
	Sex × time	*0.0001	*<0.0001
		Left PLG	Right PLG
Model	ChiSq (*P* < .0001)	489.86	343.97
	−2 log-likelihood	1200.1	1108.5
Effects (*P*)	Sex	0.0611	*0.031
	Time	*<0.0001	*<0.0001
	Sex × time	*<0.0001	*0.0001

The model fit is indicated by −2 × log-likelihood as calculated by SAS (higher indicates better fit). The *P* values for each variable are shown (italics and asterisk indicate *P* ≤ 0.05). Sex × time interaction and time were significant (*P* < 0.05) in all cases. (See also Additional files 1 and 2 for models with age and age interaction effects)

Additional analyses are shown Additional file 1 (age-related models) and Additional file 2 (right-handed only models). These files illustrate the significance comparisons via color-coded cells. Inclusion of age-affected finings only in the left MSG, with a change from non-significant to significant of sex in two models (2 and 3 in the “Additional analyses: age effects and handedness” section). Inclusion of age by time interactions affected only the left PSG, with a change from significant to non-significant of time and time by sex interaction in two models (3 and 4 in the “Additional analyses: age effects and handedness” section). Inclusion of age by sex interactions affected all right gyri, with a change from significant to non-significant of sex in two models (4 and 5 in the “Additional analyses: age effects and handedness” section). Analysis of right-handed only subjects resulted in mostly similar findings. Considering the original model (1 in the “Additional analyses: age effects and handedness” section), a change from non-significant to significant in the effect of sex appeared in the left MSG, with the other 29 model effects being unchanged. The remaining right-handed models showed 14 of 200 effects with significance changes (full details in Additional file 2).

### fMRI responses: anterior-posterior organization

**Fig. 4:**
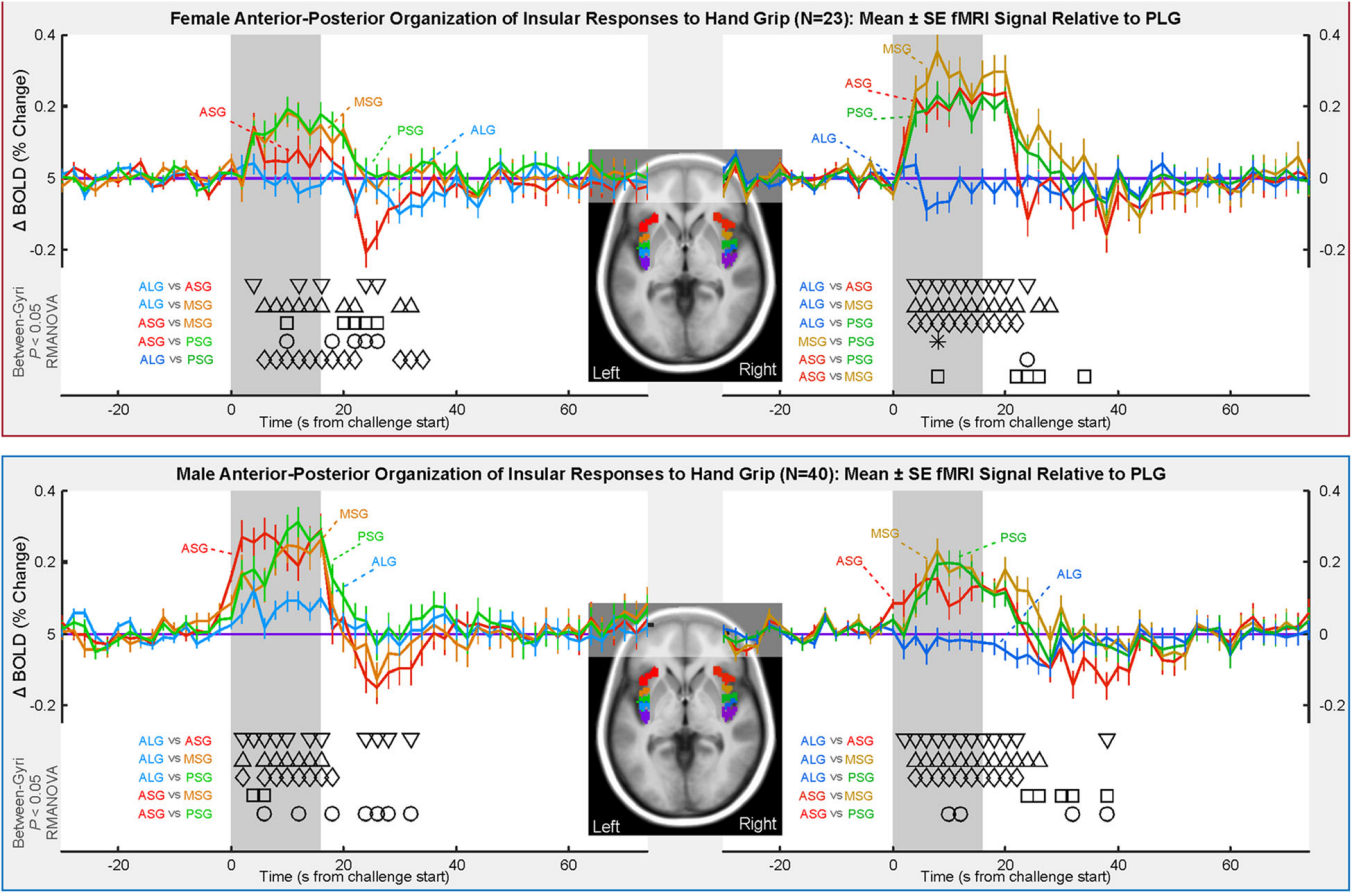
Anterior-to-posterior organization of insula fMRI responses over four static handgrip exercise challenges, illustrated by time trends relative to pattern in posterior-most gyrus (PLG). Females in *top* and males in *bottom*. Time points of between-gyrus differences are indicated by symbols above the *x*-axis and below the graphs (RMANOVA *P* < 0.05; Table 2)

In the right insular gyral responses relative to the right PLG, both female and male responses in the anterior gyri (ASG, MSG, and PSG) showed a higher response from 2 to 4 s into the challenge until 4–6 s into the recovery period (Fig. 4). In the left gyri (relative to the left PLG), in females, MSG > ALG and PSG > ALG from 6 s through the remainder of the challenge period. The left PSG response remained higher than the PLG response until 6 s into the recovery period. In the left gyri in males, ASG > ALG from 2 s into the challenge through the challenge, except at 12 s into the challenge. MSG > ALG and PSG > ALG from 2 s into the challenge through to the end of the challenge (MSG) and 2 s into the recovery period (PSG), except at 4 s into the challenge when the PLG response peaks. The anterior gyri showed mostly similar patterns of response (Table 2).

**Table 2:** Anterior-posterior model fit. Overall model chi-square (ChiSq) was always significant (*P* < 0.0001)

Anterior vs. posterior		Gyrus, time gyrus × time	Gyrus, time Gyrus × time
		Female left	Female right
Model	ChiSq (*P* < .0001)	424.31	472.4
	−2 log-likelihood	−3139.6	−2111.2
Effects (*P*)	Gyrus	*<0.0001	*<0.0001
	Time	*<0.0001	*<0.0001
	Gyrus × time	*<0.0001	*<0.0001
		Male left	Male right
Model	ChiSq (*P* < 0.0001)	1503.42	1086.15
	−2 log-likelihood	367.1	−2072.2
Effects (*P*)	Gyrus	0.12	*0.0024
	Time	*<0.0001	*<0.0001
	Gyrus × time	*<0.0001	*<0.0001

The model fit is indicated by −2 × log-likelihood as calculated by SAS (higher indicates better fit). The *P* values for each variable are shown (italics and asterisk indicate *P* ≤ 0.05). Gyrus × time interaction was significant (*P* < 0.05) in all cases. (See also Additional files 3 and 4 for models with age and age interaction effects)

Additional analyses are shown in Additional file 3 (age-related models) and Additional file 4 (right-handed only models). These files illustrate the significant comparisons via color-coded cells. Inclusion of age by gyrus interactions affected finings in females and males in the right side only, with a change from significant to non-significant of sex in two models (4 and 5 in the “Additional analyses: age effects and handedness” section). Analysis of right-handed only subjects resulted in similar findings to all subjects across 80 effects assessed (full details in Additional file 4).

### fMRI responses: left-right organization

**Fig. 5:**
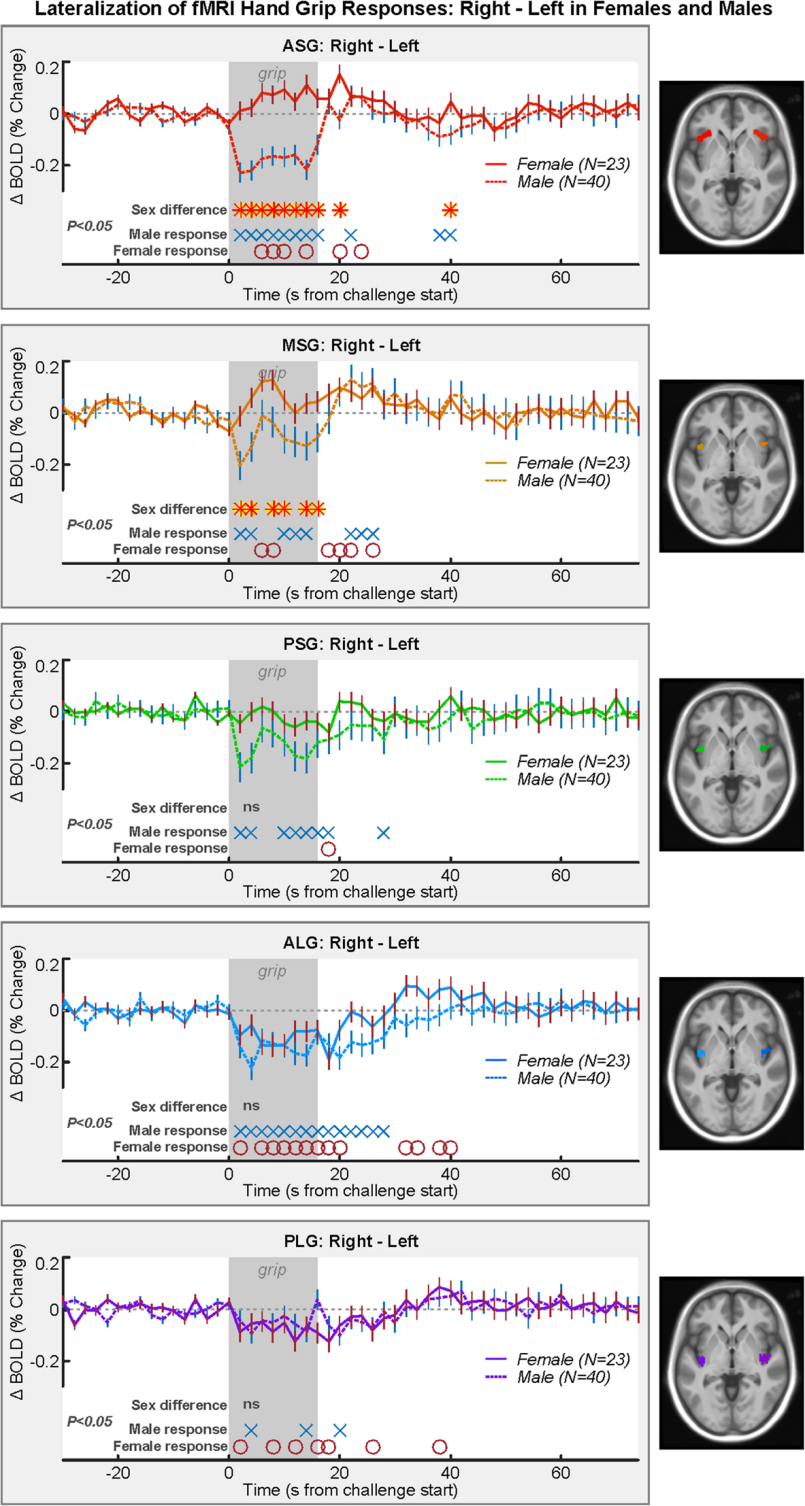
Lateralization of insula fMRI responses averaged over four static handgrip exercise challenges, illustrated by right − left time trends, such that a higher signal indicates a greater right-sided response. Time points of between-hemisphere differences in females (*red circles*) and males (*blue xs*) are indicated, as well as time points of group differences (*red-yellow asterisks*; RMANOVA *P* < 0.05; Table 3)

Lateralization of activity during the challenge was evident in the ASG for males but only sporadically for females (Fig. 5). In males, the right response was less than the left. The lateralization response in males differed from females, which was similar at the onset of the challenge, but the right side dipped less than the left side from 8 to 12 s. The males show a significantly more left-dominant response in the anterior ASG and MSG gyri but not the more posterior regions (Table 3).

**Table 3:** Female vs. male in laterality (right minus left) model fit

Right minus left female vs. male		Sex, time sex × time
ASG		
Model	ChiSq (*P* < 0.0001)	528.48
	−2 log-likelihood	−1000.7
Effects (*P*)	Sex	*0.0014
	Time	*<0.0001
	Sex × time	*<0.0001
MSG		
Model	ChiSq (*P* < 0.0001)	1026.5
	−2 log-likelihood	724.4
Effects (*P*)	Sex	0.3058
	Time	*<0.0001
	Sex × time	*0.018
PSG		
Model	ChiSq (*P* < 0.0001)	1275.17
	−2 log-likelihood	546.2
Effects (*P*)	Sex	0.2086
	Time	*0.0003
	Sex × time	0.2642
ALG		
Model	ChiSq (*P* < 0.0001)	817.6
	−2 log-likelihood	−334.2
Effects (*P*)	Sex	0.1589
	Time	*<0.0001
	Sex × time	0.1152
PLG		
Model	ChiSq (*P* < 0.0001)	373.45
	−2 log-likelihood	−735.8
Effects (*P*)	Sex	0.7959
	Time	*<0.0001
	Sex × time	0.9692

Overall model chi-square (ChiSq) was always significant (*P* < 0.0001). The model fit is indicated by −2 × log-likelihood as calculated by SAS (higher indicates better fit). The *P* values for each variable are shown; sex × time interaction and time were significant (*P* < 0.05) in all cases apart from male ASG and female MSG (italics and asterisk indicate *P* ≤ 0.05). (See also Additional files 5 and 6 for models with age and age interaction effects.)

Additional analyses are shown in Additional file 5 (age-related models) and Additional file 6 (right-handed only models). These files illustrate the significant comparisons via color-coded cells. Age did not alter effects in the PSG. Inclusion of age by time interactions affected finings in all other gyri, with a change from significant to non-significant of time in two models (3 and 4 in the “Additional analyses: age effects and handedness” section), and in the MSG changes from significant to non-significant of sex by time interactions. Inclusion of age by sex interactions affected findings in the ASG, with changes from significant to non-significant in the effects of sex in two models (4 and 5 in the “Additional analyses: age effects and handedness” section). Analysis of right-handed only subjects resulted in similar findings to all subjects across all but four of the 100 effects assessed (full details in Additional file 6). In the PSG, the time by sex interactions in the original model and in the model with age effect (2 in the “Additional analyses: age effects and handedness” section) changed from non-significant to significant. In the ALG, the time effect shifted from non-significant to significant in the models with age by time interactions (3 and 4 in the “Additional analyses: age effects and handedness” section).

### Interpretation: overview of key findings

Females and males show similarities in fMRI signal responses during and after the short static handgrip exercise challenge, with a peak early in the challenge period, followed by a return to or below baseline, and an increase to another peak upon release, then by a gradual return to baseline. However, the magnitude of signal change differed by sex in all but the left anterior-most gyrus (ASG), with males showing lower fMRI signals in all other regions during and just after the challenge. Females showed lower heart rate increases during the challenge, but males dropped SaO_2_ levels, which remained low throughout the protocol. The right insula showed an anterior dominance in both sexes, although with no distinction between the three short gyri. The left showed similar patterns, except that in females, the left ASG showed a lower response than the other short gyri. The left showed higher responses in all gyri in the males but only in the posterior gyri (ALG, PLG) in females. In fact, the two anterior-most gyri (ASG, MSG) showed greater right-sided responses in females. Thus, as with the Valsalva [23], the static handgrip exercise elicits generally similar responses in females and males, but selected areas, especially the ASG, show opposite patterns.

### Cardiovascular responses

Heart rate increases to static handgrip exercise in females were lower than males. An earlier finding in a younger sample also showed slightly higher heart rate increases (but not significantly so) in males vs. females during a 2-min static handgrip exercise at 40% of maximum grip strength, even though in contrast with our study, both groups started at equivalent resting heart rate levels [43]. In a smaller and younger sample (7 men and 6 women, mean age 25–26), a 2-min static handgrip exercise at 30% of maximum elicited a substantially greater heart rate increase in males than females [44]. Unlike females, males also greatly increased muscle sympathetic nerve activity (MSNA), although this activity occurred later in the challenge. An earlier study found no sex difference in heart rate responses to a 30% static handgrip exercise [45], but only one measure at 60 s was used, and that analysis would be less sensitive that the time-trend comparisons used in more recent studies. The combined evidence suggests that females, on average, have a reduced change in heart rate to the static handgrip exercise. Since heart rate is a significant component of cardiac output increase to a pressor challenge, females presumably have a lower need or resort to other vascular and heart control mechanisms to accommodate day-to-day perfusion challenges.

The SaO_2_ sex differences showed an enduring effect of prior static handgrip exercise challenges across the four task (~6 min) protocol. The RMANOVA performed on the averaged challenges was therefore confounded by the lack of return to baseline in such a way as to increase false negatives, but even so, SaO_2_ showed significant declines during the challenge. The whole-protocol plot shows SaO_2_ in both males and females declining around the first task and remaining low. Dips are visible in the males during the three subsequent grip periods, reflected also in the RMANOVA outcomes. However, intriguingly, the SaO_2_ declines from *before* the initial task period, about 20–30 s into the baseline, suggesting an anticipation effect. The decline is over 0.5%, which for the fMRI BOLD signal is a substantial change; typical fMRI activations are measured as signal changes around 1%. However, the BOLD effect as a response to neuronal activation is relatively independent of baseline state [46]; therefore, while blood SaO_2_ could affect the resting level of the fMRI signal, the activation should be similar.

The reason the males showed an overall lower SaO_2_ is unclear. One possibility is that males were holding their breath because they were trying harder, a phenomenon observed with other tasks [47]. Tasks deemed “masculine” such as strengthening are associated with greater effort by males [48].

### Insular function

The insula is involved in regulation of autonomic actions but also has an integrative role for body sensations, and during the static handgrip exercise, both of these functions will be represented in the fMRI signals. However, the sensory and interoceptive responses are principally located in the posterior insula [49–51], whereas the predominant responses to the static handgrip exercise were in the more anterior short gyri. Other functions associated with the insula such as pain and mood are unlikely to be represented during this short static handgrip exercise challenge.

While the anterior insula is active during many tasks involving sympathetic activation, the signal increases here show that the activity in the structure may also increase with suppression of parasympathetic action, which is accompanied by cardiovascular changes, including rising heart rate, blood pressure, and cardiac output [52]. Presumably, sympathetic outflow was not substantially increased during this brief static handgrip exercise [26], leaving, we speculate, a process dependent on suppression of parasympathetic activity. Previous neuroimaging studies on static handgrip exercise over a longer challenge period also show there is no direct relationship between MSNA and insular activation [53].

### Lateralization

The left-sided dominance in all gyri in males likely reflects a combination of parasympathetic responses associated with vagal withdrawal and contralateral representation of the right-hand sensory-motor signals. That is, parasympathetic withdrawal could involve an active process in the insula perhaps reflecting an increase in inhibition [54]. Additionally, right-handed sensori-motor representation is in left cortical brain regions, which include the insula [55]. Thus, in males, any right-sided sympathetic dominance was likely masked by left-sided representation of the hand. The concept that sympathetic action is dominated by the right side of the brain is well supported by human and animal data [56–59], as well as by the Valsalva study conducted during the same series of experiments as the present static handgrip exercise challenge [23]. An interesting complementary experiment would be a left static handgrip exercise, during which we predict a larger right-sided insular response.

In contrast, while females showed equivalent left-sided dominance in the posterior long gyri, the two anterior-most gyri showed right-sided dominance (and the mid-region, MSG, showed no lateralization). Thus, females and males showed a different response pattern organization. Considering a simple model, the findings could reflect a greater sympathetic-related activation on the right, less sensory-motor-related activation on the left, or less parasympathetic activity in the left. Since the distribution of autonomic functions is more anterior [49], the pattern is consistent with greater or lower parasympathetic-related activation in females than males. That is, if we assume that representation of limb sensations is similar in males and females across the whole insula and is primarily localized in posterior regions, the similar female-male responses in the posterior long gyri suggest no difference in the contralateral sensorimotor activation. The female static handgrip exercise pattern of higher right ASG signals is opposite to the Valsalva-induced pattern of *lower* responses in this region in females [23].

### Anterior autonomic dominance

On the right side, the three anterior short gyri showed larger responses than the two posterior long gyri, highlighting a dominant role over the posterior regions during increased cardiovascular activity, a pattern consistent with other challenges [22]. Unlike the Valsalva maneuver, the anterior, mid, and posterior right short gyri showed similar response patterns, and the anterior and posterior right long gyri were also very similar. Thus, the anterior-most ASG may have distinct functions only when strong sympathetic activation is occurring, which is not the case with the present short static handgrip exercise.

The males showed a similar pattern on both left and right, with the anterior short gyri following similar time courses and the ALG signal being close to the PLG. However, females showed only the MSG and PSG with similar increases. The left ASG patterns in females were lower than the other two short gyri for most of the grip period and remained close to those of the long gyri. Thus, the left ASG patterns in females were inconsistent with those of other short gyri in males and females on the left and right. This distinction is in contrast to the Valsalva, where the right ASG showed unique patterns of response. The present findings reinforce the difference in organization in the anterior-most gyrus of the insula, specifically in females.

### Age and handedness influences

Age differed slightly between the sexes, and age modestly influenced the findings, including some sex by age-related variation that may relate to menopausal status in females. The models with sex × age (4 and 5 in the “Additional analyses: age effects and handedness” section) altered the time effect in a consistent manner across multiple analyses: of the 19 original models, nine showed a significant group effect (5 between-sex, 3 between gyri, 1 between-sex laterality); the group effect reflects differences in fMRI signal magnitude averaged over the entire period including baseline and challenge. All of these time effects changed from significant to non-significant with the inclusion of age × sex, showing that consistent differences in average magnitude of signal responses are accounted for with inclusion of this interaction. The lack of significant intensity differences over the entire period is consistent with the normalization of the signal to a percent change from baseline (the standard fMRI approach). Only other sex-difference models significantly affected with inclusion of age factors: (1) the left MSG showed a change from non-significant to significant of the effect of sex with age as a variable (models 2 and 3 in the “Additional analyses: age effects and handedness” section) and (2) the left PSG showed a change in time and sex × time from significant to non-significant in the models with age × time (models 3 and 4 in the “Additional analyses: age effects and handedness” section). These two changes were modest, suggesting that the influences are minor.

The inclusion of only right-handed subjects did not substantially alter the pattern of results. Handedness can influence autonomic function [60] but not necessarily to a static handgrip exercise [61, 62].

### Clinical implications for patients with insular injury

Insular lesions or stroke compromises autonomic regulation [63, 64]. The findings here suggest that unilateral injury may result in dysregulation varying according to the stimulus and differ by sex. Assuming that greater fMRI activation represents a more active subregion role, a lesion or stroke in the right anterior-most insula may affect sympathetic regulation in males more than females. Similarly, a right-sided anterior insular insult could affect parasympathetic regulation in females more than males. Right-sided insular stroke leads to autonomic imbalance, but such effects have not been separated by sex [65]. Other regulatory actions such as heart rhythm and blood glucose control are also affected in people with insular stroke. Right-sided stroke is strongly associated with cardiac arrhythmias, yet there is a dearth of sex-specific data [66]. Similarly, right-sided stroke is associated with hyperglycemia [67], and sex-based metabolic issues are a particular concern. The data suggest there may be substantial sex differences in clinical consequences of insular damage.

### Future studies

The findings raise new research questions. One broad question is whether activity in the right anterior insula is closely related to sympathetic activation. A simple extension of existing experiments or a secondary analysis of longer paradigm data could address the question: does the right insula respond once sympathetic activation to the static handgrip exercise occurs (30–60 s into the challenge)? In such a longer paradigm that leads to sympathetic activation, does the insular right side show an increase? Since MSNA studies show activity from approximately 1 min into a static handgrip exercise, an fMRI analysis could look at that time period, as opposed to immediately after onset. Another question is whether the lateral representation of the hand performing the grip is strongly represented in the results. Comparison with a left static handgrip exercise, and passive motion or very low grip strength fMRI changes could allow the sensory-motor effects to be separated.

The clinical consequences of lateralized insular injury are now well established, yet the sex-specific patterns remain to be comprehensively characterized. Many of the existing datasets could be analyzed on a sex-specific basis. The findings do indicate that new projects should collect sex and hormonal information such as menopausal status.

### Limitations

The sample likely included pre- and post-menopausal women, a factor that would only indirectly be reflected in the additional models with age by sex interactions (4 and 5 in the “Additional analyses: age effects and handedness” section). We did not measure hormone status in females, which likely contributed to variability, since menopausal status and stage of the menstrual cycle influence many aspects of autonomic function [68, 69]. One study in younger people did show a slight reduction in diastolic and to a lesser extent systolic pressure in women in mid luteal vs. early follicular phase (estrogen is lower during the early follicular phase), but the effect size was many times smaller than the male female difference regardless of phase [43]. Heart rate did not show such phase-related differences in that study, suggesting hormonal status is unlikely to have been a main driver of the present findings.

Variability in individual anatomy could easily have led to variability in true separation of gyri [39, 70], but the fMRI signal itself is only sensitive to within a few millimeters, so a finer anatomical distinction would be unlikely to make a noticeable difference in the findings.

## Conclusions

Insular responses to a brief static handgrip exercise differ by sex. The magnitude of such responses is overall higher in females. The anterior-posterior distribution is similar in all but the left, anterior-most ASG in females, with all other short gyri responding similarly, with greater activation than the posterior long gyri. Lateralization in males showed a left-dominant response, which could relate to the parasympathetic withdrawal, especially in the anterior regions, as well as a somatosensory representation, particularly in the posterior regions. As with the Valsalva maneuver, the anterior ASG appears to have unique functional organization, although the left-side patterns are highlighted by pre-static handgrip exercise-induced parasympathetic withdrawal and the right-sided patterns by the Valsalva-induced strong sympathetic activation. The mechanisms underlying the sex variation specifically in this region likely relate to a combination of basal state, hormonal influences, and sex-specific brain structure.

## Additional files

Additional file 1: Between-sex differences for all models. Excel file showing global and main effects of RMANOVA for original model (left-most) and four models with age-related effects. For the three main effects in the original model (Table 1), the significance in models with age effects is classified via color-coding cells as “same” (green) or “changed” (orange) according to whether the *P* value shifted from below or above the 0.05 threshold. (XLSX 14 kb)

Additional file 2: Right-handed subjects only, between-sex differences for all models. Excel file showing global and main effects of RMANOVA for original model (left-most) and four models with age-related effects. The effects are compared with the models with all subjects (Additional file 1), and the significance classified via color-coding cells as “same” (green) or “changed” (orange) according to whether the *P* value shifted from below or above the 0.05 threshold. (XLSX 14 kb)

Additional file 3: Between-gyri differences for all models. Excel file showing global and main effects of RMANOVA for original model (left-most) and four models with age-related effects. For the three main effects in the original model (Table 2), the significance in models with age effects is classified via color-coding cells as “same” (green) or “changed” (orange) according to whether the *P* value shifted from below or above the 0.05 threshold. (XLSX 12 kb)

Additional file 4: Right-handed subjects only, between-gyri differences for all models. Excel file showing global and main effects of RMANOVA for original model (left-most) and four models with age-related effects. The effects are compared with the models with all subjects (Additional file 3), and the significance classified via color-coding cells as “same” (green) or “changed” (orange) according to whether the *P* value shifted from below or above the 0.05 threshold. (XLSX 12 kb)

Additional file 5: Between-sex differences in laterality for all models. Excel file showing global and main effects of RMANOVA for original model (left-most) and four models with age-related effects. For the three main effects in the original model (Table 3), the significance in models with age effects is classified via color-coding cells as “same” (green) or “changed” (orange) according to whether the *P* value shifted from below or above the 0.05 threshold. (XLSX 12 kb)

Additional file 6: Right-handed subjects only, between-sex differences in laterality for all models. Excel file showing global and main effects of RMANOVA for original model (left-most) and four models with age-related effects. The effects are compared with the models with all subjects (Additional file 5), and the significance classified via color-coding cells as “same” (green) or “changed” (orange) according to whether the *P* value shifted from below or above the 0.05 threshold. (XLSX 12 kb)

## Abbreviations

ALG: Anterior long gyrus (sub-region of insular cortex)
ASG: Anterior short gyrus (sub-region of insular cortex)
BOLD: Blood oxygen-level dependent (type of MRI signal, including those in fMRI scans)
MSG: Mid short gyrus (sub-region of insular cortex)
PLG: Posterior long gyrus (sub-region of insular cortex)
PSG: Posterior short gyrus (sub-region of insular cortex)
RMANOVA: Repeated measures analysis of variance
